# Real-time classification and sensor fusion with a spiking deep belief network

**DOI:** 10.3389/fnins.2013.00178

**Published:** 2013-10-08

**Authors:** Peter O'Connor, Daniel Neil, Shih-Chii Liu, Tobi Delbruck, Michael Pfeiffer

**Affiliations:** Institute of Neuroinformatics, University of Zurich and ETH ZurichZurich, Switzerland

**Keywords:** deep belief networks, spiking neural network, silicon retina, sensory fusion, silicon cochlea, deep learning, generative model

## Abstract

Deep Belief Networks (DBNs) have recently shown impressive performance on a broad range of classification problems. Their generative properties allow better understanding of the performance, and provide a simpler solution for sensor fusion tasks. However, because of their inherent need for feedback and parallel update of large numbers of units, DBNs are expensive to implement on serial computers. This paper proposes a method based on the Siegert approximation for Integrate-and-Fire neurons to map an offline-trained DBN onto an efficient event-driven spiking neural network suitable for hardware implementation. The method is demonstrated in simulation and by a real-time implementation of a 3-layer network with 2694 neurons used for visual classification of MNIST handwritten digits with input from a 128 × 128 Dynamic Vision Sensor (DVS) silicon retina, and sensory-fusion using additional input from a 64-channel AER-EAR silicon cochlea. The system is implemented through the open-source software in the jAER project and runs in real-time on a laptop computer. It is demonstrated that the system can recognize digits in the presence of distractions, noise, scaling, translation and rotation, and that the degradation of recognition performance by using an event-based approach is less than 1%. Recognition is achieved in an average of 5.8 ms after the onset of the presentation of a digit. By cue integration from both silicon retina and cochlea outputs we show that the system can be biased to select the correct digit from otherwise ambiguous input.

## 1. Introduction

**Figure 1:**
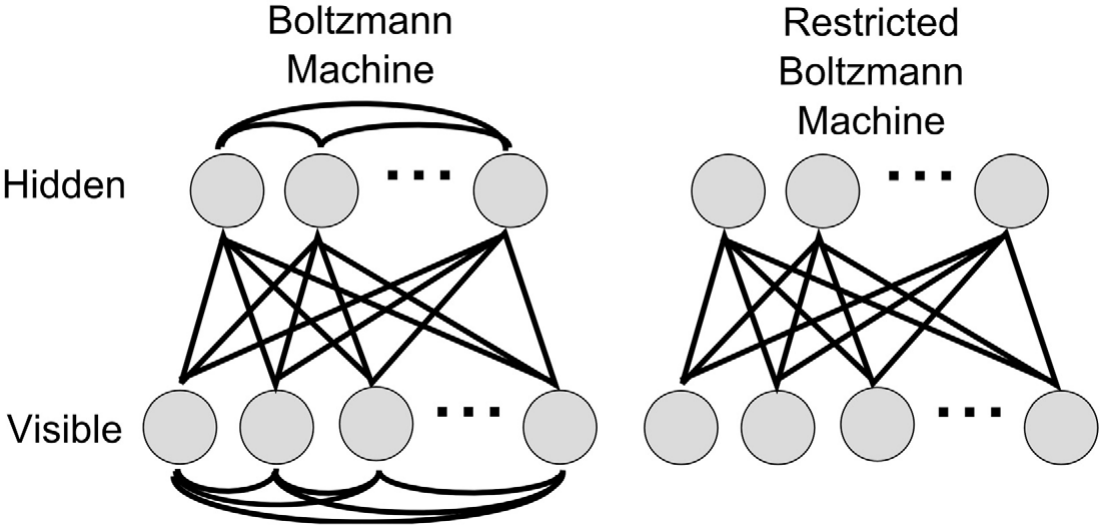
**Boltzmann and Restricted Boltzmann Machines.** A Boltzmann machine is fully connected within and between layers, whereas in a RBM, the lateral connections in the visible and hidden layers are removed. As a result, the random variables encoded by hidden units are conditionally independent given the states of the visible units, and vice versa.

Deep Learning architectures, which subsume convolutional networks (LeCun et al., 1998), deep autoencoders (Hinton and Salakhutdinov, 2006), and in particular DBNs (Bengio et al., 2006; Hinton et al., 2006; Hinton and Salakhutdinov, 2006) have excelled among machine learning approaches in pushing the state-of-the-art in virtually all relevant benchmark tasks to new levels. In this article we focus on DBNs, which are constructed as hierarchies of recurrently connected simpler probabilistic graphical models, so called Restricted Boltzmann Machines (RBMs). Every RBM consists of two layers of neurons, a hidden and a visible layer, which are fully and symmetrically connected between layers, but not connected within layers (see Figure 1). Using unsupervised learning, each RBM is trained to encode in its weight matrix a probability distribution that predicts the activity of the visible layer from the activity of the hidden layer. By stacking such models, and letting each layer predict the activity of the layer below, higher RBMs learn increasingly abstract representations of sensory inputs, which matches well with representations learned by neurons in higher brain regions e.g., of the visual cortical hierarchy (Gross et al., 1972; Desimone et al., 1984). The success of Deep Learning rests on the unsupervised layer-by-layer pre-training with the Contrastive Divergence (CD) algorithm (Hinton et al., 2006; Hinton and Salakhutdinov, 2006), on which supervised learning and inference can be efficiently performed (Bengio et al., 2006; Erhan et al., 2010). This avoids typical problems of training large neural networks with error backpropagation, where overfitting and premature convergence pose problems (Hochreiter et al., 2001; Bengio et al., 2006). The data required for pre-training does not have to be labeled, and can thus make use of giant databases of images, text, sounds, videos, etc. that are now available as collections from the Internet. An additional attractive feature is that the performance of deep networks typically improves with network size, and there is new hope of achieving brain-like artificial intelligence simply by scaling up the computational resources.

With the steady increase in computing power, DBNs are becoming increasingly important for an increasing number of commercial *big data* applications. Using gigantic computational resources industry leaders like Google or Microsoft have started to invest heavily in this technology, which has thus been recently named one of the Breakthrough Technologies of 2013 (MIT Technology Review, 2013), and has led to what has been called the “second reNNaissance of neural networks” (Ciresan et al., 2010). This is the result of the success stories of Deep Learning approaches for computer vision (Larochelle et al., 2007; Lee et al., 2009; Ciresan et al., 2010; Le et al., 2012), voice recognition (Dahl et al., 2012; Hinton et al., 2012; Mohamed et al., 2012), or machine transcription and translation (Seide et al., 2011; MIT Technology Review, 2013). Despite this potential, the sheer number of neurons and connections in deep neural networks requires massive computing power, time, and energy, and thus makes their use in real-time applications e.g., on mobile devices or autonomous robots infeasible. Instead of speculating on Moore's law to achieve progress through faster and cheaper computing resources in the future, we argue that fast and energy efficient inference in DBNs is already possible now, and is an ideal use case for neuromorphic circuits (Indiveri et al., 2011), which emulate neural circuits and event-based, asynchronous communication architectures in silicon. This is motivated by the fact that in the brain, having many neurons and connections is not a factor that constrains the processing time, since all units operate in parallel, and only the arrival of spike events triggers processing, so the neural circuits can adapt the processing speed to the rate at which input spikes occur. This scheme would allow the system to remain silent, consuming little power, in potentially long silent periods, and still allow fast recognition when bursts of input activity arrive, a scenario that is realistic for natural organisms. These advantages have been recently realized for event-based convolutional networks using convolution chips (Camuñas Mesa et al., 2010; Farabet et al., 2012), but a principled way of building DBNs models out of spiking neurons, in which both feed-forward and feed-back processing are implemented has been lacking.

This paper presents the first proof-of-concept of how to transform a DBN model trained offline into the event-based domain. This allows exploiting the aforementioned advantages in terms of processing efficiency, and provides a novel and computationally powerful model for performing recognition, sampling from the model distribution, and fusion of different sensory modalities. Although our current implementation is in software, and not on neuromorphic VLSI, inference with small DBNs runs in real time on a standard laptop, and thus provides the first necessary step toward the goal of building neuromorphic hardware systems that efficiently implement deep, self-configuring architectures. In particular, the novel framework allows us to apply state-of-the-art computer vision and machine learning techniques directly to data coming from neuromorphic sensors that naturally produce event outputs, like silicon retinas (Lichtsteiner et al., 2008) and cochleas (Liu et al., 2010).

Our main contribution is a novel method for adapting conventional CD training algorithms for DBNs with spiking neurons, using an approximation of the firing rate of a Leaky Integrate-and-Fire (LIF) spiking neuron (Siegert, 1951; Jug et al., 2012). After training with a time-stepped model, the learned parameters are transferred to a functionally equivalent spiking neural network, in which event-driven real-time inference is performed. In this article we explicitly perform learning of the network offline, rather than with spike-based learning rules, but note that there is a high potential for future event-driven DBNs that could exploit spike-timing based learning for recognizing dynamical inputs. We evaluate the spiking DBNs by demonstrating that networks constructed in this way are able to robustly and efficiently classify handwritten digits from the MNIST benchmark task (LeCun et al., 1998), given either simulated spike-train inputs encoding static images of digits, or live inputs from neuromorphic vision sensors. In addition we present an event-based DBN architecture that can associate visual and auditory inputs, and combine multiple uncertain cues from different sensory modalities in a near-optimal way. The same architecture that is used for inference of classes can also be used in a generative mode, in which samples from the learned probability distribution are generated through feed-back connections.

The aspect of combining feed-back and feed-forward streams of information is an important deviation from traditional purely feed-forward hierarchical models of information processing in the brain (Van Essen and Maunsell, 1983; Riesenhuber and Poggio, 1999), and DBNs provide a first step toward linking state-of-the-art machine learning techniques and modern models of Bayesian inference and predictive coding in the brain (Rao and Ballard, 1999; Hochstein and Ahissar, 2002; Friston, 2010; Markov and Kennedy, 2013). The importance of recurrent local and feed-back connections in the cortex seems obvious from the anatomy (da Costa and Martin, 2009; Douglas and Martin, 2011; Markov et al., 2012), and *in vivo* experiments (Lamme et al., 1998; Kosslyn et al., 1999; Bullier, 2001; Murray et al., 2002), but the precise role of feed-back processing is still debated (Lamme et al., 1998; Bullier, 2001; Kersten and Yuille, 2003). One hypothesized role is in multisensory integration, and as generative Bayesian models, DBNs are very well suited to perform such tasks, e.g., by combining visual and auditory cues for improved recognition (Hinton et al., 2006). We will thus discuss the potential impact of DBNs as abstract functional models for cortical computation and learning.

The structure of this article is as follows: The mathematical framework and the algorithms used for training and converting conventional DBNs into spiking neural networks are presented in Section 2. Section 3 shows the application of the framework to simulated spike train inputs and real visual and auditory inputs from neuromorphic sensors. Implications of this new framework are discussed in Section 4.

## 2. Materials and methods

### 2.1. Deep belief networks

A DBN (Bengio et al., 2006; Hinton et al., 2006) is a multi-layered probabilistic generative model. The individual layers consist of simpler undirected graphical models, so called *Restricted Boltzmann Machines* (RBMs), typically with stochastic binary units. A RBM has a bottom layer of “visible” units, and a top layer of “hidden” units, which are fully and bidirectionally connected with symmetric weights. The difference between standard Boltzmann machines and RBMs is that in the restricted model units within the same layer are not connected (see Figure 1), which makes inference and learning within this graphical model tractable. The visible layers of RBMs at the bottom of a DBN are clamped to the actual inputs when data is presented. When RBMs are stacked to form a DBN, the hidden layer of the lower RBM becomes the visible layer of the next higher RBM. Through this process, higher level RBMs can be trained to encode more and more abstract features of the input distribution.

In a binary RBM the units stochastically take on states 0 or 1, depending on their inputs from the other layer. Denoting the states of visible units with *v*_*i*_, the states of hidden units with *h*_*j*_, the weights connecting these units with *w*_*ij*_, and the biases of visible and hidden units with *b*^(*v*)^_*i*_ and *b*^(*h*)^_*j*_ respectively, a RBM encodes a joint probability distribution *p*(**v, h | θ**), defined via the energy function

(1) $$E(v,h;\theta )=-\sum_{i}\sum_{j}w_{ij}v_{i}h_{j}-\sum_{i}b_{i}^{\left(v\right)}v_{i}-\sum_{j}b_{j}^{\left(h\right)}h_{j},$$

where **θ** = (**w, b**^(*v*)^, **b**^(*h*)^). The encoded joint probability can then be written as

(2) $$p(v,h|\theta )=\frac{\exp(-E(v,h;\theta ))}{\sum_{v^{\prime }}\sum_{h^{\prime }}\exp(-E(v^{\prime },h^{\prime };\theta ))}.$$

From equations (1) and (2) the following stochastic update rules for the states of units were derived (Hinton and Sejnowski, 1986), such that on average every update results in a lower energy state, and ultimately settles into an equilibrium:

(3) $$p(v_{i}=1|h,\theta )=\sigma \left(\sum_{j}w_{ij}h_{j}+b_{i}^{\left(v\right)}\right)$$

(4) $$p(h_{j}=1|v,\theta )=\sigma \left(\sum_{i}w_{ij}v_{i}+b_{j}^{\left(h\right)}\right),$$

where σ(*x*) = 1/(1 + exp(−*x*)) is the sigmoid function, and the units will switch to state 0 otherwise. When left to run freely, the network will generate samples over all possible states (**v, h**) according to the joint probability distribution in (2). This holds for any arbitrary initial state of the network, given that the network has enough time to become approximately independent of the initial conditions.

#### 2.1.1. Training a RBM

During learning, the visible units are clamped to the actual inputs, which are seen as samples from the “data distribution.” The task for learning is to adapt the parameters θ such that the marginal distribution *p*(**v** | **θ**) = ∑_**h**_ *p*(**v, h | θ**) becomes maximally similar to the true observed data distribution p^*^(**v**), i.e., the log-likelihood of generating the observed data needs to be maximized. Hinton et al. (2006) have shown that this gradient ascent on the log-likelihood w.r.t. the weights *w*_*ij*_ can be efficiently approximated by a Gibbs-sampling procedure, which alternates between stochastically updating the hidden and visible units respectively. For the RBM this leads to the learning rule

(5) $$\Delta w_{ij}=\eta \left({\langle v_{i}h_{j}\rangle }_{\text{data}}-{\langle v_{i}h_{j}\rangle }_{\text{model}}\right),$$

where 〈. 〉_data_ denotes an average over samples with visible units clamped to actual inputs, 〈. 〉_model_ denotes an average over samples when the network is allowed to sample all units freely, and η is the learning rate.

Using a sampling approximation normally requires creating enough samples such that the network can settle into an equilibrium. However, for a RBM the CD algorithm (Hinton et al., 2006) has been developed, which uses only a single sample for the data and model distribution, and performs very well in practice. CD first samples new values for all hidden units in parallel, conditioned on the current input, which gives a complete sample (**v**_data_, **h**_data_) for the data distribution. It then generates a sample for the visible layer, conditioned on the hidden states **h**_data_ sampled in the first step, and then samples the hidden layer again, conditioned on this new activity in the visible layer. This generates a sample (**v**_model_, **h**_model_) from the model distribution. The weight update can then be computed as

(6) $$\Delta w_{ij}=\eta \left(v_{i,\text{data}}h_{j,\text{data}}-v_{i,\text{model}}h_{j,\text{model}}\right).$$

#### 2.1.2. Persistent CD and transient weights

Since the form of sampling induced by CD strongly biases the samples from the model distribution toward the most recently seen data, one can alternatively use so-called Persistent Contrastive Divergence (Tieleman, 2008). In this approach, the model distribution is initialized arbitrarily, and at every iteration of the training process further samples are created by sampling conditioned on the most recently sampled hidden states, which are maintained between data points.

There is a delicate balance between sampling and learning in Persistent CD: Although fast learning is generally desirable, too fast learning can result in too fast changes of the encoded joint probability distribution, which can cause the equilibrium distribution to change too fast for the Markov chain of model states to ever settle in. Nevertheless, high learning rates have turned out to be beneficial in practice, since they increase the mixing rates of the persistent Markov chains (Tieleman and Hinton, 2009). Following the suggestions in Tieleman and Hinton (2009) we used so called “fast weights,” which are added to the regular weights of the network, and decay exponentially with each training step. When sampling from the model distribution, the fast weights are updated with the rule:

(7) $$\Delta w_{ij}^{\text{fast}}=-\alpha {\langle v_{i}h_{j}\rangle }_{\text{model}}.$$

We will later show that such transient weight changes can be interpreted as short-term plasticity in a spiking neural network implementation.

#### 2.1.3. Constructing DBNs by stacking RBMs

As discussed previously, DBNs can be constructed by stacking RBMs and interpreting the hidden layer of the lower RBM as the visible layer of the next layer. It has been shown that adding hidden layers and applying the previously discussed unsupervised learning methods for RBMs is guaranteed to increase the lower bound on the log-likelihood of the training data (Hinton et al., 2006). Higher layers will tend to encode more abstract features, which are typically very informative for classification tasks. The top-layer of the DBN can then be trained with supervised learning methods, and the whole multi-layer network can be optimized for the task through error backpropagation (Hinton and Salakhutdinov, 2006; Hinton et al., 2006).

DBNs can also be used for associating different sets of inputs, e.g., from different sensory modalities. In this case one can build pre-processing hierarchies for both inputs independently, and then treat the top layers of these hierarchies as a common visible layer for a new association layer on top of them (Hinton et al., 2006). DBNs are therefore not necessarily single hierarchies, but can also exhibit tree-like architectures.

### 2.2. Discrete-time and event-driven neuron models

Traditional RBMs are, like most machine-learning models, simulated in time-stepped mode, where every neuron in a layer gets updated at every time step, and the size of this time step Δ*t* is fixed throughout the simulation. While training is typically easier to achieve with continuous and time-stepped neuron models, the event-driven model has the potential to run faster and more precisely. This is because the states of LIF neurons in the event-based network are only updated upon the arrival of input spikes, and only at these times the neurons decide whether to fire or not. Temporal precision is limited only by the numerical representation of time in the system (as opposed to the duration of the time-step parameter). A drawback is that not all neuron models, e.g., smooth conductance-based models, can be easily converted into event-driven models.

In the standard formulation (Hinton et al., 2006), units within RBMs are binary, and states are sampled according to the sigmoidal activation probabilities from Equations (3) and (4). We call such neuron models sigmoid-binary units. In Nair and Hinton (2010) it was shown that an equivalent threshold-linear model can be formulated, in which zero-mean Gaussian noise 
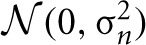
 with variance σ^2^_*n*_ is added to the activation functions:

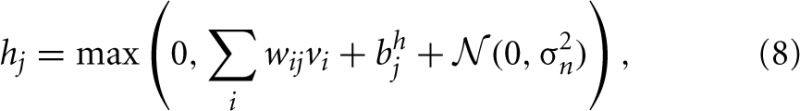



and similarly for the sampling of visible units.

A threshold-linear function can also be used to approximate the expected firing rates of simplified spiking neurons under constant current stimulation, such as the LIF neuron (Gerstner and Kistler, 2002), which is one of the simplest, yet biologically relatively plausible models for spiking neurons. In this model each incoming event adds to the membrane potential *V*_*m*_ according to the strength *w*_*ij*_ of the synapse along which the event occurred. Incoming spikes within an absolute refractory period *t*_*ref*_ after an output spike are ignored. Spikes are generated deterministically whenever the membrane potential crosses the firing threshold *V*_*th*_, otherwise the membrane potential decays exponentially with time constant τ. Simple versions of LIF neurons can be simulated in an event-based way, since membrane potentials only need to be updated upon the arrival of input spikes, and spikes can only be created at the times of such input events. For a LIF neuron representing *h*_*j*_, which receives a constant input current *s*_*j*_ = ∑_*i*_ *w*_*ij*_ *v*_*i*_ corresponding to the weighted sum of inputs from connected visible units, the expected firing rate ρ_*j*_(*s*_*j*_) is:

(9) $$\rho_{j}(s_{j})=\{\begin{array}{ll} {\left(t_{ref}-\tau \log\left(1-\frac{V_{th}}{s_{j}}\right)\right)}^{-1} & \text{if }s_{j}\geq V_{th}\\ 0 & \text{otherwise} \end{array}$$

The above equation holds when the neuron is injected with a constant input, but under realistic conditions the neuron receives a continuous stream of input spike trains, each arriving to first approximation as samples from a Poisson process with some underlying firing rate. For this case, a more accurate prediction of the average firing rate can be obtained using **Siegert neurons** (Siegert, 1951; Jug et al., 2012). Siegert neurons have transfer functions that are mathematically equivalent to the input-rate output-rate transfer functions of LIF neurons with Poisson-process inputs. In order to compute the Siegert transformation for a neuron receiving excitatory and inhibitory inputs with rates $\left(\vec{\rho_{e}},\vec{\rho_{i}}\right)$ and weights $\left(\vec{w_{e}},\vec{w_{i}}\right)$ respectively, we first have to compute the auxiliary variables

$$\begin{array}{cccccc} \mu_{Q} & = & \tau \sum (\vec{w_{e}}\vec{\rho_{e}}+\vec{w_{i}}\vec{\rho_{i}}) & \sigma_{Q}^{2} & = & \frac{\tau }{2}\sum (\vec{w_{e}^{2}}\vec{\rho_{e}}+\vec{w_{i}^{2}}\vec{\rho_{i}})\\ ϒ & = & V_{rest}+\mu_{Q} & \Gamma & = & \sigma_{Q}\\ k & = & \sqrt{\tau_{syn}/\tau } & \gamma & = & \left|\zeta (1/2)\right| \end{array}$$

where τ_*syn*_ is the synaptic time constant (for our purposes considered to be zero), and ζ is the Riemann zeta function. Then the average firing rate ρ_*out*_ of the neuron with resting potential *V*_*rest*_ and reset potential *V*_*reset*_ can be computed as (Jug et al., 2012):

(10) $$\begin{array}{l} \rho_{out}=(t_{ref}+\frac{\tau }{\Gamma }\sqrt{\frac{\pi }{2}}\cdot \\ \;\int_{V_{reset}\;+\;k\gamma \Gamma }^{V_{th}\;+\;k\gamma \Gamma }{\exp\left[\frac{{\left(u-ϒ\right)}^{2}}{2\Gamma^{2}}\right]\cdot \left[1+\text{erf}\left(\frac{u-ϒ}{\Gamma \sqrt{2}}\right)\right]du)}^{-1}. \end{array}$$

A RBM trained using Siegert units can thus be easily converted into an equivalent network of spiking LIF neurons: By normalizing the firing rate in Equation (10) relative to the maximum firing rate 1/*t*_*ref*_, ρ_*out*_ can be converted into activation probabilities as required to sample RBM units in Equations (3, 4) during standard CD learning with continuous units. After learning, the parameters and weights are retained, but instead of sampling every time step, the units generate Poisson spike trains with rates computed by the Siegert formula Equation (10).

### 2.3. Training the network

#### 2.3.1. Task

The network was trained on a visual classification task on the MNIST benchmark dataset for machine learning (LeCun et al., 1998). This set consists of a collection of 28 × 28 gray-scale images of handwritten digits, of which 60,000 form a training set, and 10,000 an independent test set. In order to make the network more robust, we modified the training set by adding small random translations (±15%), rotations (±3°) and scalings (±10%). The modified training set contains 120,000 images.

#### 2.3.2. Network architecture

**Figure 2:**
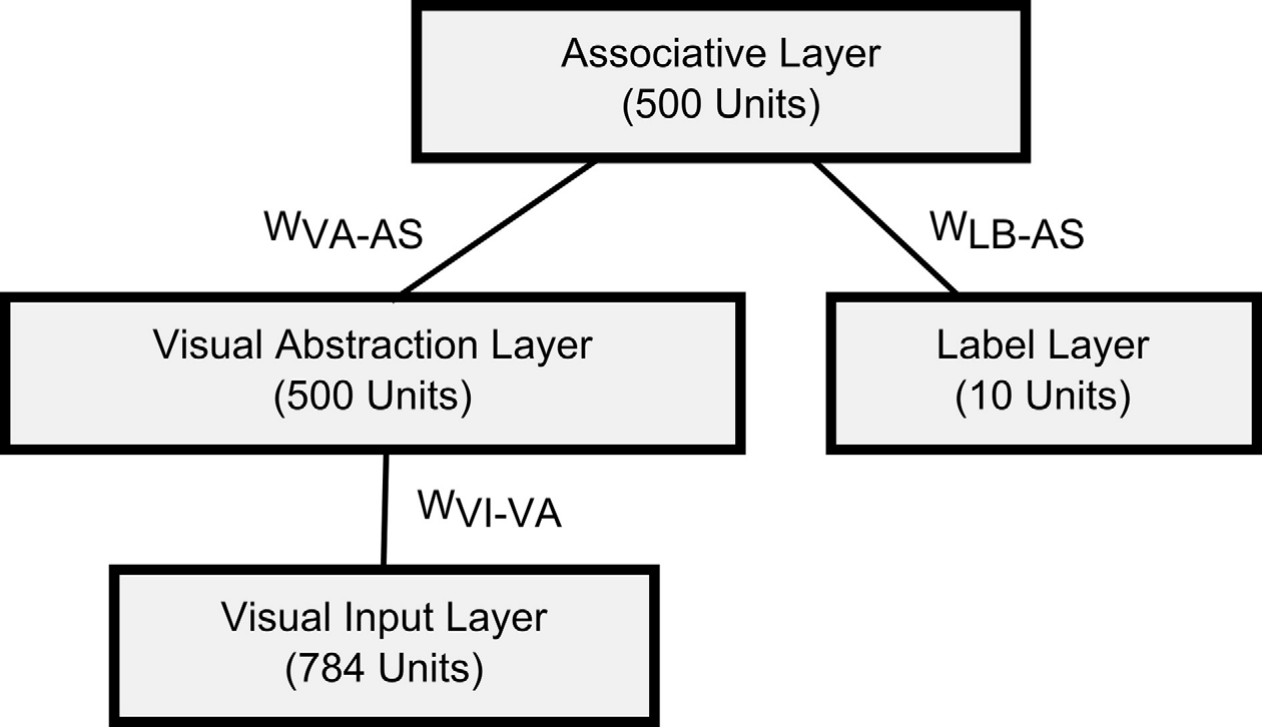
**Architecture of the DBN for handwritten digit recognition.** The connections between layers represent the weights of a RBM.

For the visual classification task we trained a DBN with one input layer of 784 visual input units (corresponding to the pixels of 28 × 28 input images), a 500-unit “Visual Abstraction Layer,” a 500-unit “Association Layer,” and a 10-unit “Label Layer,” with units corresponding to the 10 digit-classes. The architecture of the network is shown in Figure 2. Since our goal in this article is to demonstrate a proof-of-concept for spiking DBNs, the 785-500-500-10 network we used is substantially smaller than the 784-500-500-2000-10 network used previously for the MNIST task (Hinton et al., 2006), or the state-of-the-art network in Ciresan et al. (2010).

#### 2.3.3. Training

Each RBM in Figure 2 was first trained in a time-stepped mode with Siegert neurons as individual units, for which we fixed the parameters for resting and reset potential, membrane time constants, and refractory period. Since the output rates of Siegert neurons are not constrained to the interval [0, 1] like in Sigmoid-Binary units, the outputs were normalized, such that the maximum possible firing rate (given by 1/*t*_*ref*_) had a value of 1. As training algorithm for RBMs we applied persistent Contrastive Divergence learning (Hinton et al., 2006) and the fast weights heuristics described in Section 2.1.2. We also applied a modification to the training process proposed by Goh et al. (2010) to encourage sparse and selective receptive fields in the hidden layer.

Learning proceeded in a bottom-up fashion, starting by training the weights between the Visual Input and the Visual Abstraction Layers. Next, the weights of the Associative Layer were trained, using input from the previously trained Visual Abstraction Layer and the supervised information in the Label Layer as the joint visible layer of the RBM. For each layer we trained for 50 iterations over the complete training set.

### 2.4. Simulation of an event-driven DBN

We created simulators for arbitrary event-driven DBNs in Matlab and Java. The simulation can be either run in *Recognition mode*, where input is applied at the bottom layer, and the label has to be inferred through bottom-up processing, or in *Generation mode*, where the activity of the label layer is fixed, and the network samples activity in the Visual Input Layer through top-down connections, according to the learned generative model. Bottom-up and top-down processing can also be activated simultaneously.

In *Recognition mode*, the DBN is shown a number of test images, which are transformed into spike trains that activate the Visual Input Layer. A Poisson spike train is created for each pixel with a rate proportional to the pixel intensity, and all firing rates are scaled such that the total input rate summed over all 28 × 28 pixels is constant (between 300 and 3000 spikes per second). The goal is to compute the correct classification in the Label Layer. For every input image, the input activations are sampled as Poisson spike trains with rates proportional to the pixel intensities. Classification can be done in one of two ways: first, we can turn on only bottom-up connections from the Visual Input Layer toward the Label Layer, and observe which of the neurons in the Label Layer spikes the most within a fixed time interval. The second variant is to use only bottom-up connections between Visual Input and Visual Abstraction Layer, but activate all recurrent connections in the other RBMs. Information about previous inputs is stored both within the membrane potentials and the recurrent spiking activity within the network. Recognition is thus achieved through a modulation of the persistent network activity by input spike trains. In the absence of input, the network will continue to be active and drift randomly through the space of possible states according to the encoded generative model.

**Figure 3:**
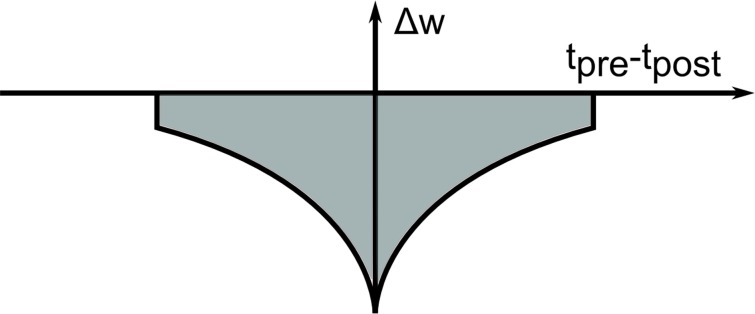
**Short-term plasticity kernel for Generation mode.** The “fast-weight” STDP kernel temporarily depresses all synapses in which the pre- and post-synaptic neurons were active shortly after each other, depending on the spike timing difference *t*_*pre*_ − *t*_*post*_. As a result, the network is constantly being pushed out of its present state.

This principle is exploited in the *Generation mode*, where units within the Label Layer are stimulated, and activation propagates recurrently through the top-level RBM, and top-down to the Visual Input Layer. Thus, analyzing these samples from the generative model provides a way to visualize what the network has learned so far. If the DBN is activated in this way, it might settle into a particular state, but could become stuck there, if this state corresponds to a local minimum of the Energy landscape according to (1). This can be avoided by using a short-term depressing STDP kernel in Generation mode, which temporarily reduces the weights of synapses where pre- and post-synaptic neurons are active within the same short time window (see Figure 3). These short-term modifications vanish over time, and the weights return to their original values. This modification is inspired by the idea of using auxiliary “fast-weights” for learning (Tieleman and Hinton, 2009), which transiently raise the energy of any state that the network is currently in, thereby slightly pushing it out of that state. The effect is that the network, instead of settling into an energy well and remaining there, constantly explores the whole space of low-energy states. This is a useful feature for search and associative memory tasks, where the network represents a cost function through the encoded energy landscape, and the task is to converge to a maximally likely state starting from an arbitrary initial state, e.g., an incomplete or ambiguous input. We demonstrate this in Section 3.4 in the context of multi-sensory integration.

### 2.5. Real-time implementation

#### 2.5.1. Neuromorphic visual input

We developed a real-time variant of the event-driven DBN which receives inputs from neuromorphic sensors. Visual input was obtained from the DVS (Lichtsteiner et al., 2008), an event-generating image sensor consisting of 128 × 128 pixels, which asynchronously outputs streams of address events in response to local relative light-intensity changes. The events are tagged with the address of the creating pixel, a time-stamp, and an ON or OFF polarity tag, which indicates whether the event was created in response to an increase or decrease of light intensity over that pixel. Events are transmitted via a USB port to a computer, and processed in the open-source jAER software framework written in Java (Delbruck, 2013). The networks were first trained in Matlab, and then transferred into the jAER software, where they could run in real-time in response to event stream inputs. We did not use the polarity information for our purposes, and down-sampled the 128 × 128 pixels to a resolution of 28 × 28, which matched the resolution of the images in the MNIST training set. These events were fed into the Visual Input Layer (see Figure 2) while the DVS was moved by hand across several hand-drawn images.

#### 2.5.2. Multi-sensory fusion

We also created a task in which visual stimuli from a silicon retina and auditory stimuli from a silicon cochlea (see Section 2.5.3) were associated with each other in real-time. During training the presentation of a pure tone was always paired with the presentation of an image of a handwritten digit. Table 1 shows the tones and frequencies that were used, and the visual-auditory pairing scheme. The network thus had to learn to associate the two sensory domains, e.g., by resolving ambiguity in one sensory stream through information from the other stream.

**Table 1:** **Paired tones and digits in multi-sensory fusion task**.

**Tone**	**A**_**4**_	**B**_**4**_	**C**_**5**_	**D**_**5**_	**E**_**5**_	**F**_**5**_	**G**_**5**_**#**	**A**_**5**_	**B**_**5**_	**C**_**6**_
Freq.(Hz)	440.0	493.9	523.3	587.3	659.3	698.5	830.6	880.0	987.8	1046.5
Digit	0	1	2	3	4	5	6	7	8	9

During training pure tones with given frequencies (upper rows) were paired with an associated digit (bottom row).

The DBN architecture for sensory fusion is described in detail in Section 3.4 and shown in Figure 8.

#### 2.5.3. Neuromorphic auditory input

Auditory input was received from the AER-EAR2 (Liu et al., 2010) neuromorphic auditory sensor, which was built to mimic the biological cochlea. The device transforms input sounds into streams of spikes in 64 channels responsive to different frequency ranges. We found that since spikes of the silicon cochlea tend to be phase-locked to the sound waveform to which they are responding, the distribution of Inter-spike Intervals (ISIs) was a more precise indicator of the frequency of pure input tones than the distributions of channels from which the spikes originated. We preprocessed the auditory spikes with an event-based ISI histogramming method wherein 100 ISI bins were distributed logarithmically between 0.833 and 2.85 ms (350–1200 Hz), and for each bin an input LIF unit was assigned which was stimulated every time an ISI occurred on any channel that was within the unit's designated frequency-range. The output events of these units were then routed to the Auditory Input Layer (see Section 3.4 and Figure 8).

As stimuli we chose the pure tones from Table 1 from the A-minor harmonic scale, ranging from A4 (440 Hz) to C6 (1046.5 Hz), which were played for 1 s each into the silicon cochlea. We recorded the spike response of neurons in the Auditory Input Layer, which fired whenever enough input events from AER-EAR2 in their ISI range were received. For training in the time-stepped domain we constructed data vectors for auditory data by computing the average firing rates of Auditory Input Layer neurons over time bins of 100 ms, evaluated every 30 ms.

## 3. Results

This section presents the classification performance, shows the generative mode of operation, and presents sensor fusion experiments. For the results in sections 3.1 and 3.2 we use simulated spike-train input (see Section 2.4). Inputs from neuromorphic sensors (Section 2.5) are directly used in the results of sections 3.3 and 3.4.

### 3.1. Classification performance

**Figure 4:**
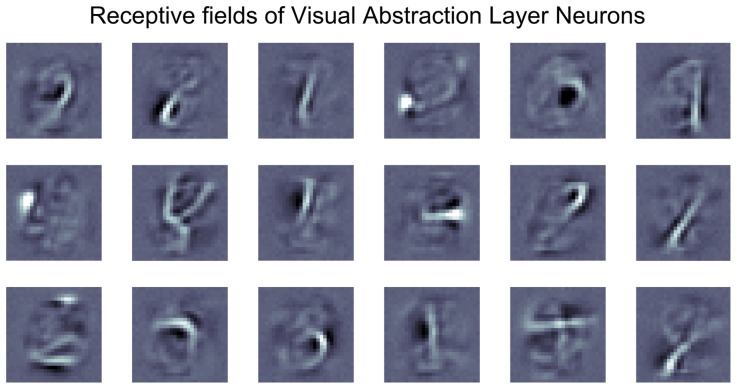
**Analysis of weights learned in the DBN.** Visualization of the weights learned by a subset of neurons in the Visual Abstraction Layer for 28 × 28 images in the MNIST task. Each image shows the vector of weights feeding into one neuron.

Three variants of DBNs were trained, using the architecture shown in Figure 2 for the MNIST visual classification task: the first two variants are time-stepped models using sigmoid-binary or Siegert neurons respectively (see Section 2.2), the third is an event-driven DBN using LIF neurons that were converted from Siegert neurons used during training. The networks were all trained in time-stepped mode for 50 iterations over the modified 120,000 example MNIST dataset using a variant of Contrastive Divergence learning (see Section 2.3). Figure 4 shows the features learned by a subset of the neurons in the RBM for the Visual Abstraction Layer. One can see that this first layer has learned through unsupervised learning to extract useful features for the discrimination of handwritten digits, in this case parts of digits. The classification performance shown in Table 2 was evaluated on images from the MNIST test set, using simulated Poisson spike trains with a total rate of 300 spikes per second for the whole image as input for event-based models. The size of our DBN is substantially smaller than in current state-of-the-art deep network approaches for MNIST, e.g., (Ciresan et al., 2010), but Table 2 shows that the performance is in a very good range (above 94%). More importantly for this proof-of-concept study, the performance loss when switching to spiking neuron models is small (on the order of 1%), and can possibly be further improved when going to larger network sizes.

**Table 2:** **Classification performance on the MNIST test set for two time-stepped and one event-based LIF neuron model**.

**Neuron model**	**Domain**	**% correct**
Sigmoid-Binary	time-step	97.48
Siegert	time-step	95.2
LIF	event-based	94.09

Inputs for the event-based model were simulated Poisson spike trains (see Section 2.4).

### 3.2. Generation mode

In *Generation mode* the network does not receive external input at the bottom layers. Instead one of the top layers (in our case the Label Layer in Figure 2) is stimulated, and activity spreads in top-down direction through the network. This provides a way to visualize what has been learned in the probabilistic generative model encoded in the bi-directional weights.

Since the network is event-driven, and neurons fire only upon the arrival of input spikes, an initial stimulus in at least one of the layers is needed to push the network from a silent state into one of self-sustaining activity, provided that the neuron parameters and recurrent connectivity allow this. We performed empirical exhaustive parameter search over firing thresholds *V*_*th*_ and membrane time constants τ in a fully trained network of LIF neurons and measured the mean firing rate within the network after 1 s of 100 Hz stimulation of one Label Layer unit, and 5 s without external stimulation. This allowed us to identify parameter regimes that allow self-sustained activity of about 20 Hz average activity in *Generation mode* (τ = 800 ms, *V*_*reset*_ = 0, *V*_*th*_ = 0.005).

**Figure 5:**
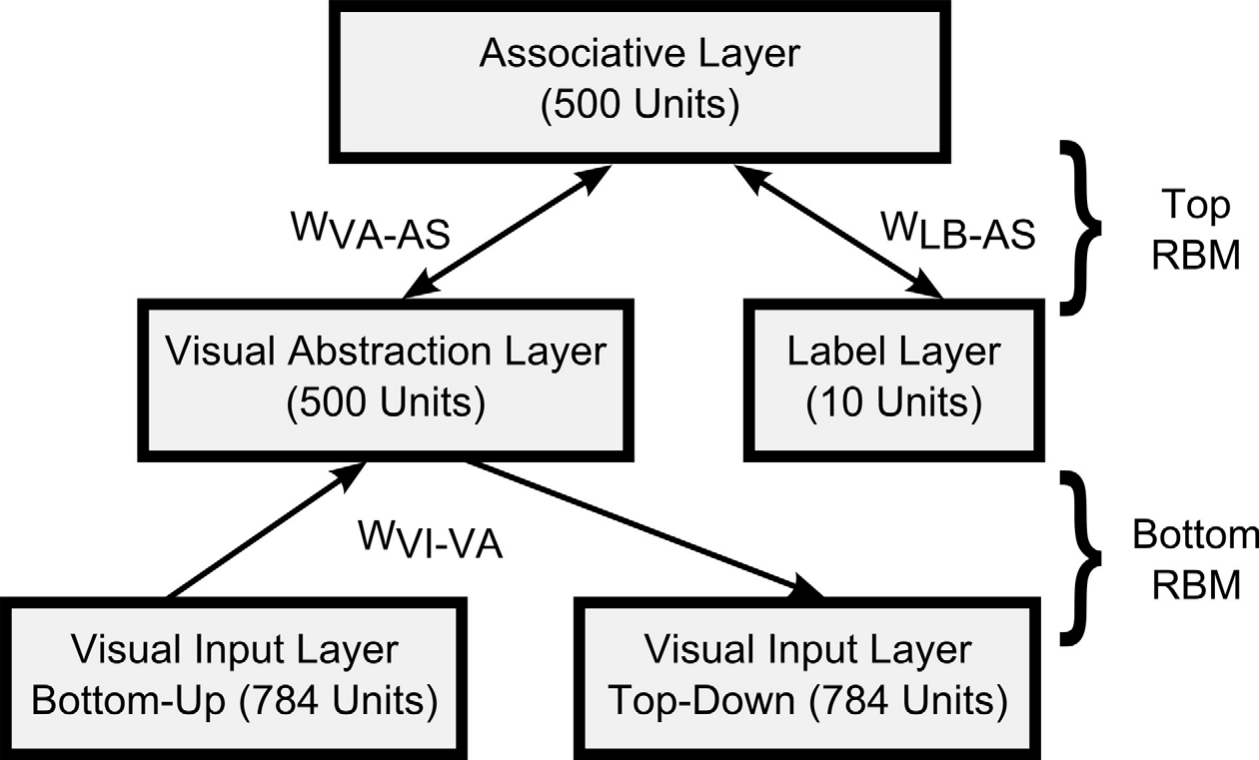
**DBN architecture for recognition and generation.** The Visual Input Layer was split into a bottom-up and a top-down part, used for projecting inputs in Recognition mode, or visualizing top-down activity in Generation mode.

To visualize the activity of the DBN in Generation mode we modified the architecture in Figure 2 that was used for training on the MNIST dataset. In the new architecture shown in Figure 5 the lowest layer is split up after training into two Visual Input Layers, one projecting only bottom-up from inputs to the Visual Abstraction Layer, and another copy that is purely driven by top-down connections. The weight matrices for bottom-up and top-down connections are identical. Thus, the top layers of the network form the recurrent model that encodes the data distribution, whereas the bottom part either projects inputs through bottom-up connections in Recognition mode, or visualizes the activity of the top layers through top-down connections in Generation mode. If both bottom-up and top-down connections are activated at the same time, the top-down Visual Input Layer visualizes a processed image of what the network ‘believes’ it is seeing in the bottom-up Visual Input Layer. This process performs probabilistic inference by which evidence from the current input is combined with the prior distribution over likely MNIST images encoded in the DBN weights, and a posterior estimate of the most likely input is generated.

**Figure 6:**
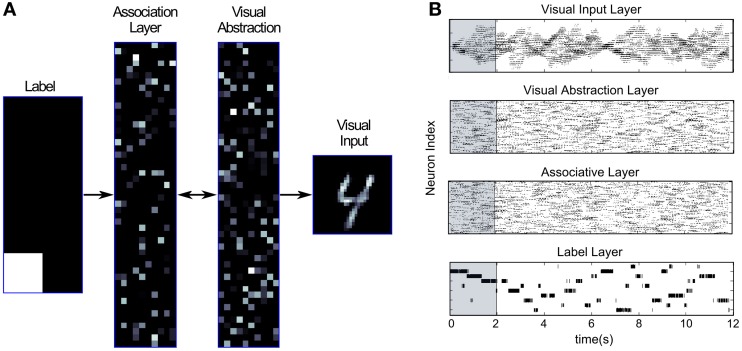
**Generation mode of the event-driven DBN. (A)** Screen capture of the network while generating samples of input activations corresponding to class “4.” The neuron corresponding to label “4” was stimulated in the Label Layer (left), and activity propagated through the whole network. The snapshot shows a single example of activity in the Visual Input Layer (right) that is sampled from the generative model encoded in the weights of the DBN. Through short-term depressing synapses (see Figure 3) the network starts to drift through the space of all potential compatible input activations. **(B)** Raster plot of the DBN in Generation mode. The Label Layer (bottom) is initially stimulated for 2 s (shaded region) to fire in sequence for digits 1, 2, and 3. Afterwards, the network freely samples from the encoded generative model. Although activity in the Label Layer jumps between digits, activity in the Visual Input Layer transitions smoothly.

Figure 6A illustrates the generation of samples from the encoded probabilistic model after activating a unit in the Label Layer. This induces spiking activity in the intermediate Associative and Visual Abstraction Layer, and ultimately stimulates units in the top-down Visual Input Layer, which can be visualized. Figure 6A shows the response of the network when the label unit corresponding to the class “4” is stimulated. The snapshot shows the induced activity in the lower layers, and one can clearly see that the response in the Visual Input Layer resembles closely the handwritten digits in the MNIST set that were used for training. By using short-term depressing synapses as described in Section 2.1.2 and in Figure 3 the network not just samples one single example of a “4,” but iterates through different variations that are compatible with the variance over inputs in the learned generative model. This can be best seen in Video 1 of the supplementary material.

Figure 6B shows the spiking activity in the different layers of the network in generation mode, both during a forced stimulation, and in a free self-sustained mode. The network is initially stimulated for 2 s by forcing firing of neurons in the Label Layer corresponding to digit classes “1,” “2,” and “3” (shaded region). One can see that through the recurrent connectivity activity spreads throughout the layers of the network. After 2 s the input to the Label Layer is turned off, and the network is allowed to freely generate samples from the encoded probability distribution. We can see that in the Label Layer the network jumps between different digits, whereas in the other layers, more smooth transitions are found. Switches between visually similar digits (e.g., 4 and 9) occurred more often on average than between very different digits (e.g., 0 and 1).

### 3.3. Real-time visual recognition

For this task the event-driven DBN was connected to a neuromorphic vision sensor, the 128 × 128 pixel DVS (Lichtsteiner et al., 2008). Events indicating local light intensity changes are used as inputs in the bottom-up Visual Input Layer. The whole system works in real-time, i.e., while the DVS is recording visual input, the DBN simultaneously computes the most likely interpretation of the input. By splitting up the connections between Visual Input and Visual Abstraction Layer into a bottom-up and a top-down pathway as in Figure 5 we can simultaneously classify the input in real-time, and also visualize in the top-down Visual Input Layer the interpretation of the input after recurrent processing in the DBN.

**Figure 7:**
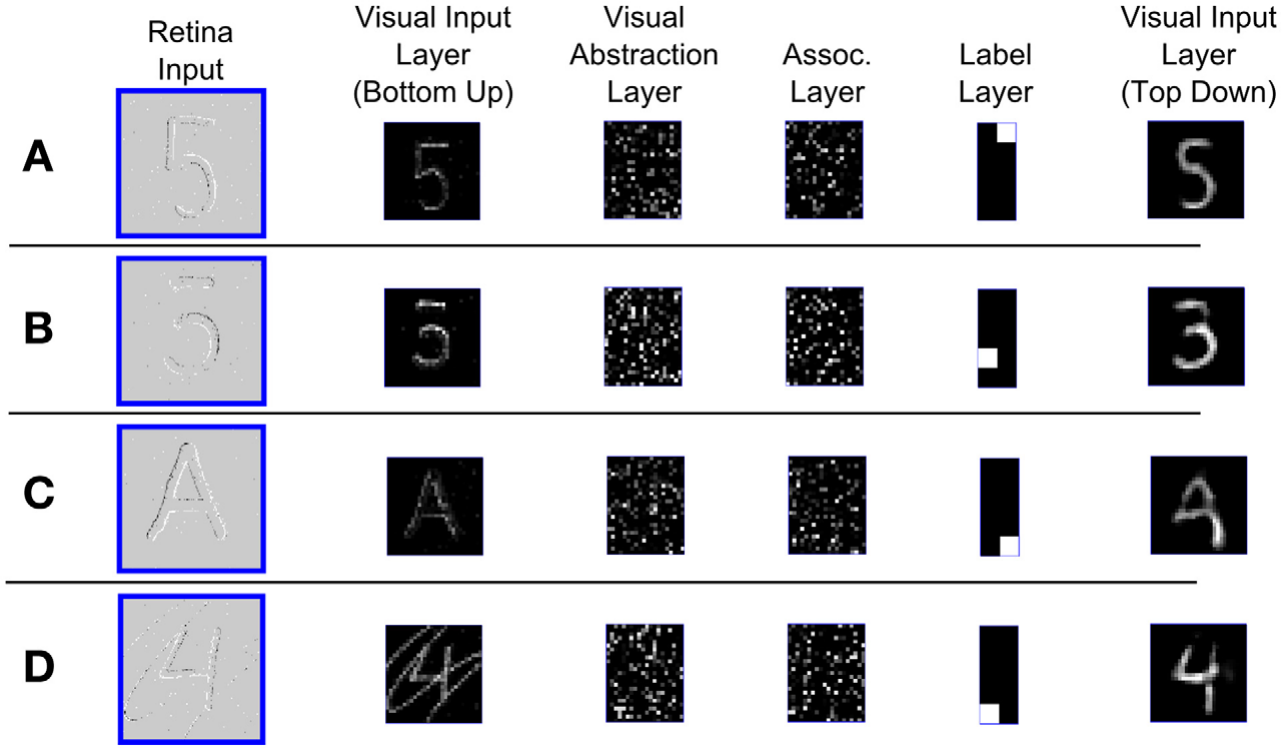
**Screen captures of the real-time spiking DBN in operation during visual handwritten digit recognition.** Each row displays a snapshot of the activity in the different layers of the network (see Figure 5) for a different visual input recorded with the DVS (left column). Neurons in the Label Layer (column 5) are arranged such that the first column represent classes 0–4 (top to bottom), and the second column classes 5–9. The rightmost column shows the top-down reconstruction of the Visual Input Layer. **(A)** The network recognizes the digit 5. **(B)** For an ambiguous input, the network alternates between the two possible interpretations “3” and “5.” The top-down reconstruction shows the current interpretation. **(C)** For an unfamiliar input (letter “A”), the network classifies it as the closest resembling digit class “9,” and reconstructs a mixture between the actual input and the generative model for class “9.” **(D)** For an input containing a distractor, the network still classifies it as the most likely input, and reconstructs an image without the distractor.

The real-time system runs as a filter in the jAER software package (Delbruck, 2013) on a standard laptop, after training the weights of the DBN offline in Matlab on the MNIST database. Figure 7 shows snapshots of the activity within the different layers of the network during operation on various stimuli recorded in real-time with the DVS. In Figure 7A the DVS was moved over a hand-drawing of the digit “5” which was not included in the training set. The left panel shows the input into the Visual Input Layer. The digit was correctly classified as a “5” in the Label Layer. On the right we can see the reconstruction of the image, which closely resembles the actual input. In Figure 7B an ambiguous input was presented, which can either be interpreted as a “3” or a “5.” The network iterated between both interpretations, in this snapshot the reconstruction on the right shows that the network currently interprets the input as a “3,” adding the missing parts of the input to match the actual shape of a digit. In Figure 7C the network is shown an input from an unknown input class, namely the letter “A.” Since the generative model learned in the network knows only digits, it classifies the input as the most similar digit, in this case “9,” and reconstructs the input as a mixture between the actual DVS input and the entrained model of the digit. In Figure 7D a digit “4” with a distracting stimulus on top was shown. It was correctly classified and reconstructed in the top-down Visual Input Layer without the distracting stimulus.

In general, the network recognized reliably all tested classes of handwritten digits in real-time, even in the presence of strong distractors, with slightly rotated images, or variations in scale or translation of the image. It can also do so very quickly: at a typical low-rate input firing rate of 3000 input spikes per second over the whole image, the DBN submits its first correct guess of the output label within an average of 5.8 ms after the onset of the simulated Poisson spike train input. Firing rates in the intermediate layers are higher, resulting in 58800 spikes/s in the 500 neuron Visual Abstraction Layer (see Figure 2), 147600 spikes/s in the 500 neuron Association Layer, and 1800 spikes/s in the 10 neuron Label Layer.

### 3.4. Real-time sensory fusion

**Figure 8:**
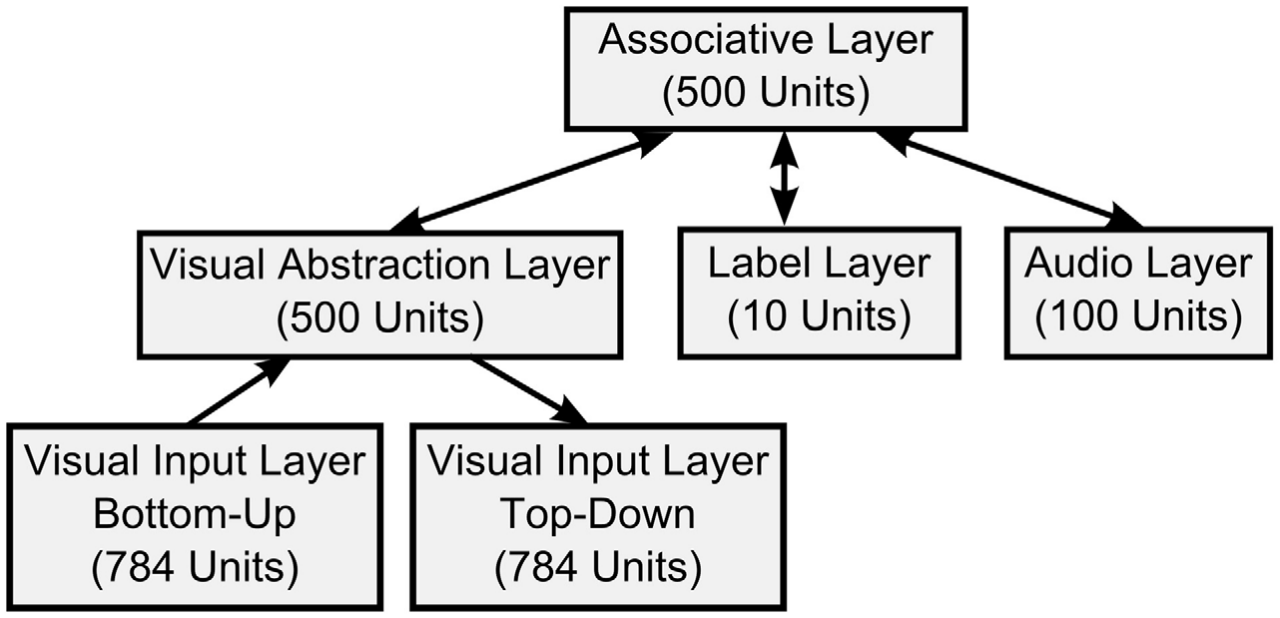
**DBN architecture of the multi-sensory fusion network.** In addition to the architecture for visual recognition in Figure 5 the Auditory Input Layer is bidirectionally connected to the top-level Association Layer. Thus, associations between visual inputs, auditory inputs, and classification results in the Label Layer can be learned during training, and classification can be achieved in real-time.

We trained a DBN to associate visual stimuli from a silicon retina, and auditory stimuli from a silicon cochlea, in order to classify them in real-time by integrating both input streams. Table 1 shows the respective association of digit images recorded with the DVS (Lichtsteiner et al., 2008), and tones of different frequencies recorded with the AER-EAR2 silicon cochlea (Liu et al., 2010). We used the DBN architecture shown in Figure 8, in which a bidirectional connection between the top-level Association Layer and the Auditory Input Layer is added.

During training a network of Siegert neurons were presented with input images from the MNIST database and pre-recorded activations of Auditory Input Layer neurons in response to the tones in Table 1 (see Section 2.5.3). After the training phase, the DBN was converted into an event-driven DBN as described previously, which was run in real-time in the jAER software package.

**Figure 9:**
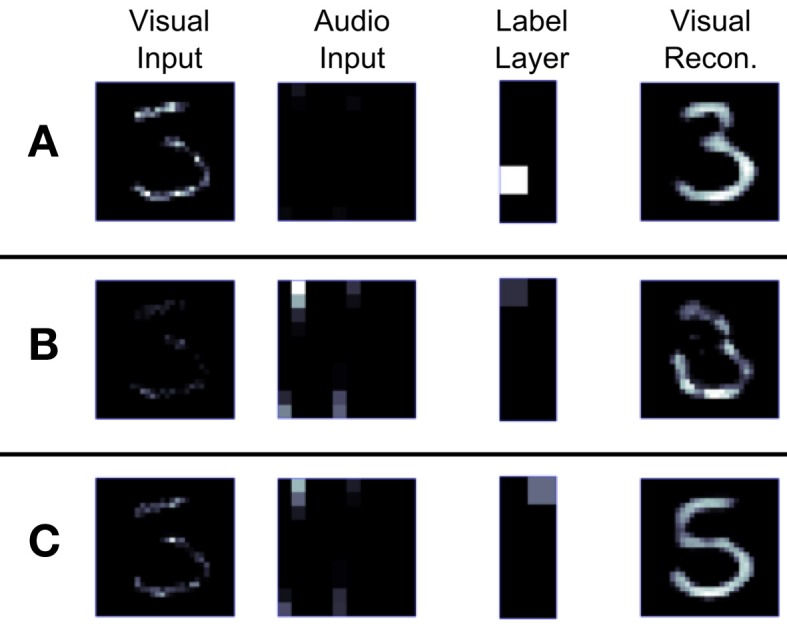
**Cue integration with a multi-sensory spiking DBN. (A)** When presenting only an ambiguous visual input to the DVS, the network in the absence of auditory input will alternate between recognizing a “3” or a “5” (see also Figure 7). **(B)** When presenting only an ambiguous auditory input to the cochlea, the network in the absence of visual input will alternate between recognizing a “0” or a “5.” **(C)** By combining the two inputs (mixing at 50%), the network reliably classifies the two ambiguous patterns as class “5,” which is the only consistent interpretation.

One key aspect of sensory fusion is the ability to integrate multiple, possibly noisy or ambiguous cues from different sensory domains to decide on the actual state of the world. We tested this by providing simultaneous visual and auditory stimuli to the DBN, such that the combination of both stimuli would provide more conclusive evidence of the true label than the single modalities. The auditory stimulus was a mixture of *A*_4_ and *F*_5_ tones corresponding to “0” and “5” digits, with four times as many input spikes corresponding to class “0” as to class “5”. Thus, if given only the audio input, the DBN should identify a “0”. Conversely, the visual input shows an ambiguous input that is consistent with either a “3” or a “5,” but very unlikely for a “0”. Figure 9 demonstrates the audio-visual fusion using an ambiguous visual input and the auditory input favoring class “0.” However, while each input stream favors an incorrect interpretation of either “3” or “0,” class “5” is correctly chosen as the most consistent representation for the combined visual-auditory input stream.

**Figure 10:**
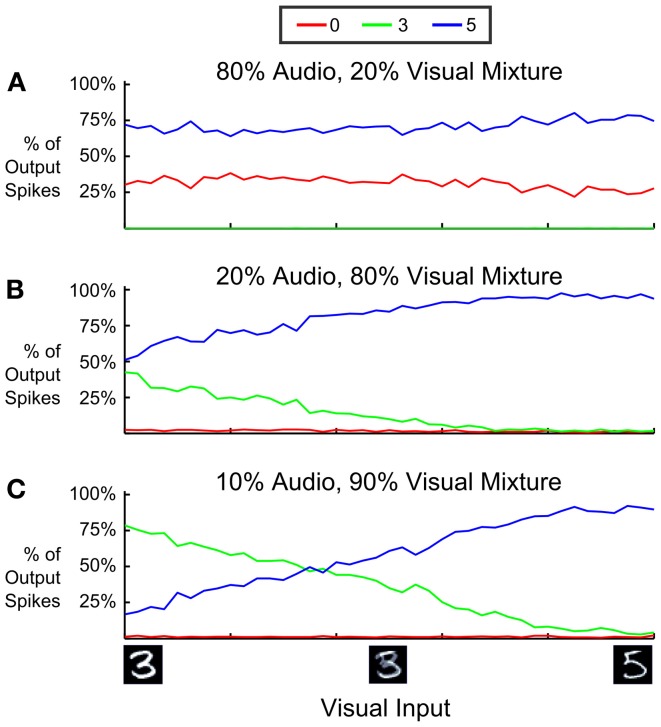
**Proportion of output spikes for 3 different mixture ratios of auditory and visual input in a multi-sensory spiking DBN.** Red, green, and blue encode the ratio of 0, 3, and 5 choices (spikes) relative to the total number of spikes emitted from the Label Layer (averaged over 10 trials). The horizontal axis sweeps the probability that visual input spikes are chosen from either a “3” digit or an aligned “5” digit. Auditory input consists of a mixture of “0” and “5” inputs, with four times more spikes indicating a “0”. Over a wide range of mixture values, the network correctly infers the only consistent interpretation of the multi-modal input, which is class “5”. Inputs that are inconsistent with the dominating sensory domain (“3” in **A**, “0” in **B,C**) are mostly suppressed.

In Figure 10 we analyzed how this depends on the relative strength of visual and auditory input streams and the ambiguity of the visual input by (1) changing the relative proportion of input spikes coming from the audio stream, and (2) interpolating the visual input between an image showing “3” and another one showing “5.” We varied the mixture of firing rates of input neurons such that 80% (Figure 10A), 20% (Figure 10B), and 10% (Figure 10C) of all input spikes came from the auditory stream, and measured the proportion of output spikes for the three classes “0,” “3,” and “5”. In panels **A** and **C** the classes that are inconsistent with the dominating auditory respectively visual input are almost completely suppressed, and class “5” is favored. One can also see from the difference between Figures 10A,B that an increase of a few spikes favoring an alternative interpretation can dramatically adjust the output choice: In this case 10% more of spikes favoring the interpretation “5” are enough to bias the classification toward the interpretation consistent with both visual and auditory input over a wide range of visual ambiguity.

## 4. Discussion

The great potential of DBNs is widely recognized in the machine learning community and industry (MIT Technology Review, 2013). However, due to the high computational costs, and the capability to integrate large amounts of unlabeled data that is freely available on the web, applications so far have strongly concentrated on big data problems (Le et al., 2012). Surprisingly little effort has gone into making this technology available for real-time applications, although the scenarios in which DBNs excel, e.g., visual object recognition, speech recognition, or multi-sensory fusion, are extremely important tasks in fields like robotics or mobile computing. An exception is the work of (Hadsell et al., 2009), who use small and mostly feed-forward deep networks for long-range vision in an autonomous robot driving off road. In general, previous attempts to reduce the running time have mostly attempted to restrict the connectivity of networks (Lee et al., 2008; Le et al., 2012), e.g., by introducing weight-sharing, pooling, and restricted receptive fields. In speech processing on mobile phones, data is first communicated to a central server where it is processed by a large DBN before the result is sent back to the mobile device (Acero et al., 2008). Online and on-board processing would be very important for mobile applications where such communication infrastructure is not available, e.g., for exploration robots in remote areas, underwater, or other planets, but this requires fast and efficient processing architectures, that conventional DBNs currently cannot provide.

We think that this presents a big opportunity for neuromorphic engineering, which has always pursued the goal of providing fast and energy efficient alternatives to conventional digital computing architectures for real-time brain-inspired cognitive systems (Indiveri et al., 2011). Here we have presented a novel method how to convert a fully trained DBN into a network of spiking LIF neurons. Even though we have only shown a proof-of-concept in software, this provides the necessary theoretical framework for an architecture that can in the future be implemented on neuromorphic VLSI chips, and first experiments in this direction are promising. The event-driven approach can be energy efficient, in particular since the required processing power depends dynamically on the data content, rather than on the constant dimensionality of the processed data. Furthermore, as we have shown, spiking DBNs can process data with very low latency, without having to wait for a full frame of data, which can be further improved if individual units of the DBN compute in parallel, rather than updating each unit in sequence. This advantage has been recognized for many years for feed-forward convolutional networks, in which almost all operations can be efficiently parallelized, and has led to the development of custom digital hardware solutions and spike-based convolution chips (Camuñas Mesa et al., 2010; Farabet et al., 2012), which through the use of the Address Event Representation (AER) protocol, can also directly process events coming from event-based dynamic vision sensors (Lichtsteiner et al., 2008). For such architectures (Pérez-Carrasco et al., 2013) have recently developed a similar mapping methodology between frame-based and event-driven networks that translates the weights and other parameters of a fully trained frame-based feed-forward network into the event-based domain, and then optimizes them with simulated annealing. In comparison, this offers increased flexibility to change neuronal parameters after training, whereas our method uses the accurate Siegert-approximation of spike rates already during the training of a bi-directional network, and does not require an additional optimization phase. The advantages of spike-based versus digital frame-based visual processing in terms of processing speed and scalability have been compared in Farabet et al. (2012), where it was also suggested that spike-based systems are more suitable for systems that employ both feed-forward and feed-back processing.

Although our model is event-based, the Siegert model (Siegert, 1951) does not make use of the precise timing of spikes. The basic theoretical framework of DBNs is not suitable for inputs that vary in time, and thus requires modifications to the network architecture (Taylor et al., 2007), or a transformation of inherently time-dependent inputs (Dahl et al., 2012). Learning with STDP-like rules in spiking DBNs provides an intriguing future possibility for a direct handling of dynamic inputs. In our current network, the short-time memory of previously seen inputs carried in the membrane potential of LIF neurons allows us to process inputs from asynchronous neuromorphic sensors, in which complete frames are never available (Lichtsteiner et al., 2008; Liu et al., 2010). We can therefore for the first time apply the state-of-the-art machine learning technique of DBNs directly to inputs from event-based sensors, without any need to convert input signals, and can classify the input while also completing the input signals using feed-back connections.

Feed-back connections are rarely used in models of biologically inspired vision, e.g., HMAX (Riesenhuber and Poggio, 1999), but as we show e.g., in Figure 7, feed-back and recurrency are essential for implementing general probabilistic inference, e.g., to infer missing, ambiguous, or noisy values in the input. Only in recent years have models become available that directly link spiking activity in recurrent neural networks to inference and learning in probabilistic graphical models. Nessler et al. (2013) have shown that learning via STDP in cortical microcircuits can lead to the emergence of Bayesian computation for the detection of hidden causes of inputs. They interpret spikes as samples from a posterior distribution over hidden variables, which is also the essential idea for neural sampling approaches (Büsing et al., 2011), in which spiking neurons implement inference in a Boltzmann machine via Markov Chain Monte Carlo sampling. Using clock-like waves of inhibition, Merolla et al. (2010) showed an alternative implementation of single Boltzmann machines with spiking neurons.

In biology, the precise role of feed-back processing is still debated, but the deficiencies of purely feed-forward architectures for processing the kind of clutter, occlusions, and noise inherent to natural scenes point at least to a role in modulation by attention signals, and in the integration of multiple cues, possibly from different modalities as well as memory and high-level cognitive areas (Lamme et al., 1998; Bullier, 2001; Kersten and Yuille, 2003). A proposal from Hochstein and Ahissar (2002) even suggests a reverse hierarchy for conscious vision, whereby fast feed-forward perception is used for a quick estimate of the *gist* of the scene, and for activating top-down signals that focus attention on low-level features necessary to resolve the details of the task. Such a model can explain the fast pop-out effect of image parts that violate model expectations, and also provides a model for fast learning without changes in the early sensory processing stages. This is consistent with a variety of theories that the brain encodes Bayesian generative models of its natural environment (Kersten and Yuille, 2003; Knill and Pouget, 2004). The hierarchical organization of sensory cortices would then naturally correspond to a hierarchy of prior distributions from higher to lower areas that can be optimally adapted to statistics of the real world in order to minimize surprise (Friston, 2010). Rao and Ballard (1999) suggested that inference in such hierarchical generative models could be efficiently performed through predictive coding. In this framework, feed-back connections would signal a prediction from higher to lower layers, whereas feed-forward connections would encode the error between prediction and actual input. In Rao and Ballard (1999) it was shown that such a model can account for several phenomena concerning the non-linear interaction of center and surround of receptive fields, and fMRI data support the theory by reporting reduced V1 activity when recognition-related activity in higher areas increases (Murray et al., 2002).

The framework of Bayesian generative models also provides a principled way of associating and integrating potentially uncertain cues from different sources, e.g., across sensory modalities (Knill and Pouget, 2004). It is well known that humans use all available cues for solving tasks, e.g., by using visual cues to improve their understanding of speech (Kayser and Logothetis, 2007; Stein and Stanford, 2008). Although traditional models have assumed that multi-sensory integration occurs only at higher association areas like superior colliculus (Felleman and Van Essen, 1991), feed-back connections from higher to lower areas or between sensory streams are likely to be involved in sensory fusion tasks. Recent studies have revealed the existence of anatomical connections that would enable cross-modal interactions also at lower levels (Falchier et al., 2002; Markov et al., 2012), and functional studies have provided some (but not conclusive) evidence of co-activations of early sensory areas by stimulation of different modalities [see (Kayser and Logothetis, 2007) for a review]. Integration might also be required within the same sensory modality, since e.g., the visual pathway splits up into at least two separate major ventral and dorsal streams.

All these arguments indicate that the traditional concept of sensory processing in the cortex as a feed-forward hierarchy of feature detectors with increasing levels of abstraction in higher layers (Gross et al., 1972; Van Essen and Maunsell, 1983; Desimone et al., 1984) needs to be reassessed (Markov and Kennedy, 2013). A closer look at the anatomy of intra- and inter-areal cortical connectivity reveals an abundance of feed-back and recurrent connections. Every brain area receives inputs from a large number of cortical and subcortical sources (Douglas and Martin, 2011; Markov et al., 2012), and feed-forward connections actually make up only a relatively small fraction of inputs to neurons along the hypothesized pathways (da Costa and Martin, 2009). Many studies have demonstrated feed-back effects, in which the activation or deactivation of a higher area alters activity in lower sensory areas (Lamme et al., 1998; Bullier, 2001; Murray et al., 2002), e.g., activation of V1 through a high-level cognitive process like visual imagery (Kosslyn et al., 1999).

DBN models can play an important role in capturing many of those effects, and the event-based framework presented in this article provides a model in which the dynamics and short-term memory properties of spiking neurons can be exploited for dealing with realistic input sequences, in our case coming from bio-inspired sensors. There are still plenty of open research questions, in particular concerning the integration of spike-timing based learning in the DBN framework, and the exploitation of spike-timing for dealing with sequences of inputs. This will likely require an adaptation of the simple RBM model used as the building block of DBNs, and will have to include recurrent lateral connections. Similar mechanisms for the processing of input sequences have been proposed in the framework of Hierarchical Temporal Memory (Hawkins and Blakeslee, 2004), which opens up new directions for combining machine learning approaches with cortical modeling.

### Conflict of interest statement

The authors declare that the research was conducted in the absence of any commercial or financial relationships that could be construed as a potential conflict of interest.
